# Unique Features of Pancreatic-Resident Regulatory T Cells in Autoimmune Type 1 Diabetes

**DOI:** 10.3389/fimmu.2017.01235

**Published:** 2017-09-29

**Authors:** Jingli Lu, Chaoqi Zhang, Lifeng Li, Wenhua Xue, Chengliang Zhang, Xiaojian Zhang

**Affiliations:** ^1^Department of Pharmacy, The First Affiliated Hospital of Zhengzhou University, Zhengzhou, China; ^2^Biotherapy Center, The First Affiliated Hospital of Zhengzhou University, Zhengzhou, China; ^3^Department of Oncology, The First Affiliated Hospital of Zhengzhou University, Zhengzhou, China; ^4^Department of Pharmacy, Tongji Hospital, Huazhong University of Science and Technology, Wuhan, China

**Keywords:** type 1 diabetes, regulatory T cells, pancreatic-resident regulatory T, immune suppression, non-obese diabetic mice

## Abstract

Recent progress in regulatory T cells (Tregs) biology emphasizes the importance of understanding tissue-resident Tregs in response to tissue-specific environment. Now, emerging evidence suggests that pancreatic-resident forkhead box P3^+^ Tregs have distinguishable effects on the suppression of over-exuberant immune responses in autoimmune type 1 diabetes (T1D). Thus, there is growing interest in elucidating the role of pancreatic-resident Tregs that function and evolve in the local environment. In this review, we discuss the phenotype and function of Tregs residing in pancreatic tissues and pancreatic lymph nodes, with emphasis on the unique subpopulations of Tregs that control the disease progression in the context of T1D. Specifically, we discuss known and possible modulators that influence the survival, migration, and maintenance of pancreatic Tregs.

## Introduction

Type 1 diabetes (T1D) is an autoimmune disease, during which immune homeostasis is destroyed and immune cells selectively attack pancreatic β cells. The development of T1D involves a complex crosstalk among immune cells of both the innate and adaptive immune systems (1). Of note, the dual roles of immune cells are often observed in β cell destruction depending on cell subsets, activation pathways, and immune microenvironment (2, 3). Thus, the specialized suppressive subsets that antagonize over-exuberant immune responses are important in inhibiting β cell destruction.

Regulatory T cells (Tregs) are critical regulators by performing suppressive functions through several well-established mechanisms (4). Studies in animal models, particularly in non-obese diabetic (NOD) mice, have demonstrated a strong association of Tregs and T1D. These cells deficiency in NOD mice accelerated disease progression (5). Therapies targeted to Tregs have also been reported in early phase clinical trials (6–8). However, different studies in T1D patients have reported conflicting results, with respect to the frequency or absolute numbers, as well as functional defect of Tregs from peripheral blood (9–13). As such, considering heterogeneity and diversity of circulating Tregs, do distinct subpopulations of Tregs engage as an important player and regulate the pathophysiology in the home tissue of T1D?

Actually, distinguishable Treg subsets that reside in tissues have attracted interest. Those tissue-resident Tregs exhibit specific phenotype and function in response to local cues, thereby promoting tissue homeostasis (14, 15). The specialized distribution of Tregs has provoked the assessment of how target tissue-resident Tregs control the development of diabetes. Now, it is evident that pancreatic-resident Treg subsets distinct from the Treg population that exist in peripheral blood and spleen (9, 16, 17). In this review, we focused on the unique features of Tregs residing in the target tissues, namely pancreatic tissues and pancreatic lymph nodes (PLNs), and how these special Tregs were maintained and functioned throughout diabetes progression.

## Phenotype of Tregs in Pancreatic Tissues and PLNs

Regulatory T cells constitute 10–15% of the total CD4^+^ T cell population in whole body but constitute a much higher proportion in local tissues such as visceral adipose tissue and intestinal tissue (18, 19). The question of whether proportion of pancreatic-resident Tregs has a similar tendency has been examined with NOD mice. In NOD mice, a small population of forkhead box P3 (Foxp3)^+^ Tregs (8–20% of CD4^+^ T cells) resided in pancreatic tissues, in comparison with 10–15% Tregs within CD4^+^ T cell compartment in lymphoid organs such as PLNs and spleen (20, 21). It gradually fluctuated with age and disease progression both in PLNs and pancreatic tissues (20–22). Controversial results were obtained when detecting Foxp3 transcripts (16), whose expression was important for the establishment and maintenance of Treg features (4). The study demonstrated that the levels of Foxp3 failed to be detected in the PLNs of prediabetic and diabetic mice (16). Divergent results are likely to reflect the mice with different gene manipulation (NOD mice versus Foxp3-GFP reporter mice), disease stages, timing and technology of Treg purification, and Treg subpopulation involved. It will be important to obtain a more global view of Treg proportion with age and disease development in T1D.

Human Treg in local pancreas has been studied only to a limited extent, but available evidence indicates that amount of Foxp3 expressed in CD4^+^ T cells and in CD25^bright^ T cells was similar within the PLNs of diabetic and non-diabetic donors (9). With difficult access to human tissues, these findings accomplished by experimental model are difficult to validate in humans. Therefore, the precise changes of pancreatic-resident Treg subsets need to be elucidated in T1D.

Comparative transcriptome analysis revealed that pancreatic-Tregs have quite different profile from their counterparts in spleen, including differential expression of suppressive mediators (Il10, Fgl2, and Lag3), chemokine receptors (Cxcr3 and Ccr5), and transcription factors that typically denote cell activation (Nr4a2, Fos, and Jun) (21). Although some of their major suppressive mediators such as Il10 and Lag3 also engaged in other tissue-resident Tregs, principal components analysis revealed that differential expression genes in pancreatic-Tregs involved in cell growth and proliferation (21). Transcriptional profiling studies which compared pancreatic-Tregs and effector T cells (Teffs) helped to further dissect differential roles of pancreatic-Tregs. Again, genes encoding functional molecules such as glucocorticoid-induced TNF receptor (GITR), CD103, Nrp-1, IL-10, and CTLA-4 were overexpressed, genes encoding transcription or signaling factors such as OBF-1, Tcf7, Eomesodermin, and Smad1 were underrepresented (23).

These data indicate that pancreatic-Treg populations have a tissue-specialized transcriptome, which may reflect their unique features in proliferation, retention, and function within pancreas in the context of T1D. Of note, there exists no information on the phenotypic particularities of Tregs residing in human pancreas, additional analysis of T1D patients are needed. In addition, skewing TCR repertoire from PLN of T1D patients has been reported (24), but no data demonstrated whether pancreatic Treg TCRs were involved.

## Unique Subpopulations of Tregs in Pancreatic Tissues and PLNs

### ICOS^+^ Treg Subset in Pancreatic Tissues

ICOS, a CD28 superfamily related molecule, is involved in T cell activation and survival (25, 26). Several works assessing the function of ICOS have been performed in the context of T1D. Systematic blockade of ICOS in NOD mice resulted in T1D exacerbation, and ICOS expression was dramatically reduced as the T1D progression, suggesting ICOS might play a protective role in T1D (23, 27). ICOS blockade led to an increased frequency of Teffs and a decreased Tregs in pancreatic tissues, but not in the PLN (23, 27). ICOS blockade also affected gene signatures of pancreatic-Tregs such as functional molecules IL-10, GITR, SOCS-2, Tcf7, Eomesodermin, and Myb, suggesting that ICOS blockade induced phenotype shift away from the Treg profile (23). The proportion of Treg expressing ICOS in pancreatic tissues was about 8% of CD4^+^ T cells, 50% of Foxp3^+^ Tregs, but only a small proportion in PLN (~2% of CD4^+^ T cells, ~18% of Foxp3^+^ Tregs) (27). The fact that ICOS was preferentially expressed in pancreatic-Tregs suggested their distinctive functional properties in T1D.

ICOS^+^ Tregs exhibited a much greater proliferative capacity compared with ICOS^−^Foxp3^+^ Tregs (27). And ICOS^+^ Tregs were more potent than ICOS^−^ Tregs at suppressing Teffs (27). Consistently, ICOS^−^ Tregs were unable to prevent T1D onset (27). These results implied defective function of ICOS^−^ Tregs in inflamed islets. Furthermore, irrespective of proliferative potential, pancreatic-ICOS^+^ Tregs had similar activation status with ICOS^−^ Tregs (27), suggesting that ICOS^+^ Tregs represented memory Tregs in islets. Of note, although ICOS^−^ Tregs had the capacity to develop into ICOS^+^ Tregs *via* TCR stimulation *in vitro*, those induced-Tregs did not suppress Teffs as efficiently as wild type ICOS^+^ Tregs (27). Similar findings have been reported for human Tregs that were isolated from thymus and periphery (28). Therefore, ICOS might be an intrinsic imprint that operated unique feature of Treg subsets during their development.

Functionally, IL-10 secreted by ICOS^+^ Treg contributed to their function in preventing diabetes (27). More specifically, IL-10-secreting Tregs were prone to residing in pancreas, but not in PLNs (27). Their frequency, particularly in the pancreas, dropped severely in genetic models of ICOS^−/−^ mice compared with those in WT mice (27). It seems that ICOS expression is required for IL-10 production by Tregs, especially those residing in the pancreas. It should be noted that signals delivered though ICOS have also suggested inducing IL-10 expression in other T cell subsets (29–32). In addition, ICOS^+^ Tregs have been identified in inflammatory skin, which developed from natural Tregs rather than adaptive Tregs (33). Similarly, skin ICOS^+^ Tregs expressed IL-10, as well as other proinflammatory cytokines IL-17 and IFN-γ (33). These data argue that ICOS^+^ Tregs might not be pancreatic-specific Tregs, but their accumulation in pancreas suggested pancreatic-specific environment tailored their function that might be related to contribution in T1D.

The molecular signals which controlled the survival and function of ICOS^+^ Tregs have also been clarified. ICOS^+^ Tregs were more susceptible to death after IL-2 withdrawn and resisted from apoptosis after IL-2 treatment (27). IL-2-mediated activation of the STAT5 pathway was responsible for ICOS expression (20). That might exist a possible IL-2-ICOS positive feedback loop in pancreatic-Treg homeostasis (20, 34). The interaction between IL-2 and ICOS that co-regulates ICOS^+^ Tregs need to be elucidated.

### ICOS^+^ Treg Subset in PLNs

As described above, the proportions of ICOS^+^ Treg in pancreas is significantly higher than those in PLN. However, ICOS^+^ Treg subsets in PLN typically expressed chemokine receptor CXCR3, which was not found in pancreatic Tregs (35). Transcriptional factor T-bet imprinted on PLN ICOS^+^ Tregs, but it was unknown whether these Tregs depended on T-bet for their generation in the PLN. Interesting, ICOS^+^CXCR3^+^ Tregs expressed IFN-γ in PLNs, which correlated with degree of pancreatic inflammation (35). The data suggest that PLN ICOS^+^ Tregs adopt a Th1-like phenotype that correlates with Th1-polarized inflammation in the pancreas. Similarly, it has confirmed that in response to IFN-γ, Tregs upregulated T-bet expression, which secondly promoted CXCR3 expression on Tregs and accumulated at sites of Th1 cell-mediated inflammation (36). It seems likely that CXCR3 was dedicated to controlling potential migration process in the home tissue and PLN (see below). It will be interesting to explore the origin of PLN ICOS^+^ Tregs—whether they migrate from pancreatic ICOS^+^ Tregs and accomplish their phenotypic features by local cues in PLNs, or whether they derive solely from population of lymphoid Tregs in PLNs.

### CXCR3^+^ Treg Subset in Pancreatic Tissues

CXCR3^+^ Tregs are another subpopulation that is abundant in the pancreas (making up nearly half of insulitic Tregs) but less frequent in spleen and PLNs (21). The proportions of pancreatic CXCR3^+^ Tregs were negatively correlated with the size of the islet infiltration, suggesting their roles in preventing inflammation (21). The transcription factor T-bet might contribute to the generation of CXCR3^+^ Tregs; when this factor was specifically deleted in Tregs, the expression of CXCR3^+^ Tregs was fully ablated, providing a potential mechanism through which CXCR3 expression was maintained in Tregs (21). Of note, an unusual property of pancreatic CXCR3^+^ Tregs is their capacity to control sex bias of T1D incidence in NOD mice (21). As such, a global understanding of pancreatic CXCR3^+^ Tregs in immune system could help explain sex difference in immune response and identify individuals with higher risk of immune-mediated diseases (37).

### IL-10- and TGF-β-Expressing Treg Subsets

TGF-β-expressing Treg is one of the effector subpopulations that residing in PLNs and pancreatic tissues (38). The levels of TGF-β in Tregs correlated with delay in diabetes development (38). TGF-β from Tregs acted on diabetogenic T cells *via* TGF-β-TGF-βR signals *in vivo* (38). However, T cell-specific disruption of TGF-β resulted in expansion of Tregs (39, 40), especially a significant increase in the frequency and number of Foxp3^+^ Tregs in the PLNs (41). Interestingly, TGFβRII-deficient Foxp3^+^ Tregs also expressed higher levels of T-bet and CXCR3 (41), which indicated TGF signals were dispensable for the development, maintenance, and function of pancreatic-resident Tregs (21).

In addition to TGF-β, IL-10 expression was also found in subsets of pancreatic-resident Tregs (27). IL-10, as an important immunomodulatory cytokine, is described to reduce IFN-γ in pancreatic β cells and prevent diabetes progression (42). IL-10 further promoted differentiation of IL-10-secreting Tregs, which formed a positive regulatory loop in IL-10 production and IL-10^+^ Treg induction (43). However, a recent study using national case-control data has challenged the notion of IL-10 as a protective role in T1D (44). It was suggested that IL-10 from neonatal blood was positively associated with T1D risk (44). Nonetheless, as described above, intra-islet Tregs mediated T1D protection was related to their production of IL-10 (27). In addition, the frequency of IL-10-secreting Tregs in pancreas is much greater than those in PLNs, and little IL-10 can be detected in PLN (27). The results suggested IL-10-secreting Tregs were prone to retain in pancreas in the context of T1D.

To conclude, our present knowledge indicates that there are at least three subsets can be distinguished on the basis of ICOS and CXCR3 that are resident in pancreatic tissues and PLNs; these Tregs express functional molecules such as IL-10 and TGF-β, which help to maintain immune homeostasis in the pancreas. Interestingly, pancreatic ICOS^+^ Tregs exhibit little similarity with those in PLN in terms of functional molecules expression and potential regulatory mechanisms, although these cells perform their protective effects in T1D. The diversity of Treg subsets in pancreas and PLNs allows functional diversity in response to tissue-specific environment changes. Of note, recent advances in studies of pancreatic-resident Tregs failed to confirm whether they were pancreatic-specific Tregs. Thus, the identification of markers for pancreatic-resident Tregs will be crucial for further studies on their function features in T1D.

## Factors That Regulate Tregs in Pancreatic Tissues and PLNs

### IL-2-Mediated Pancreatic-Treg Development in the Context of T1D

IL-2 signaling pathways are required for progression, functional programming, and expansion of Tregs (45, 46). Variants in the IL-2 gene predisposed to diabetes by reducing IL-2 production, which in turn impaired a feedback mechanism involving in Treg activity (47, 48). Diabetes susceptibility in NOD mice was reversed by treatment with IL-2, with increased Treg numbers and function (49, 50). Several works assessing the function of IL-2 in patients with T1D have showed only low dose of IL-2 played a protective role by inducing peripheral immune regulation (6, 51, 52). However, it is worth noting that this effect was not observed in Tregs-deficient mice (49), suggesting low-dose of IL-2 was inefficient to induce diabetes remission in the absence of naturally occurring Tregs.

Interestingly, pancreatic Tregs were more sensitive to IL-2 than their counterparts in PLNs and other sites in the context of T1D (49). IL-2 treatment increased proportion of Tregs in the pancreas of prediabetic mice; by contrast, when high percentage of pancreatic Tregs were already present in mice with new-onset diabetes, IL-2 treatment failed to increase pancreatic Treg numbers (49). It has ruled out the IL-2-mediated Treg increased was associated with proliferation. In addition, IL-2 signals promoted Treg survival (49), recruitment, and conversion of CD4^+^ T cells into Tregs (53). In addition to the effect of IL-2 on pancreatic Treg numbers, IL-2 directly induced pancreatic Treg activity by increasing expression of molecules associated with Treg function such as CD25, Foxp3, CTLA-4, ICOS, and GITR (20). This led to immune suppressive regulation in the target organ during T1D development.

Notably, IL-2 can stimulate the expansion and differentiation of cells expressing IL-2R (eg. CD8^+^ T cells, NK cells) during the immune response (54), which may lead to tissue destruction in the context of T1D. Thus, it needs to know how IL-2 can specifically and selectively target to Tregs, particularly pancreatic-residing Tregs in T1D.

### β-Cell-Specific IL-2 Targets to Pancreatic Tregs

Although systemic administration of IL-2 promoted pancreatic Tregs function in T1D, IL-2-mediated pleiotropic and potentially toxic effects were found in some cases. Therefore, IL-2 must specifically target to pancreas to boost suppressive Tregs while avoiding potential toxic effects. In view of this, Mark et al. developed β-cell-specific IL-2 delivery system, in which adenoassociated virus vector gene delivery was used to localize IL-2 expression to the islets of NOD mice (55). Consistent with systematic IL-2, β-cell-specific IL-2 was sufficient to prevent the onset of diabetes long term at late preclinical stages but failed to induce remission in recent-onset diabetic NOD mice (55). Specifically, β-cell-specific IL-2 administration preferentially affected islet Foxp3^+^ Tregs, which was characterized by a phenotypic shift toward CD62L^High^Foxp3^+^ Tregs (55). Furthermore, β-cell-specific IL-2 has the potent capacity to increase CD25 expression, but not CTLA-4, GITR, and ICOS (55). It is worth noting that long-term maintenance of pancreatic-Treg was associated with enhanced survival in β-cell-specific IL-2 treated mice, reflecting by increased expression of anti-apoptotic Bcl-2 and Bcl-xL (55). Also, β-cell-specific IL-2 did not promote persistent proliferation of Tregs in the islets (55). Functionally, CD62L^High^Foxp3^+^ Tregs had more robust suppressor function; this effect was not associated with elevated levels of IL-10 or TGF-β (55). Although β-cell-specific IL-2 exhibited functional similarity with systemic IL-2 in preventing T1D, these data suggest that targeting β cells might be clinically efficacious in retaining intra-islet Tregs function in the context of T1D.

### T-bet-Mediated Stability of Pancreatic-CXCR3^+^ Tregs

The transcription factor T-bet in Tregs promotes Th1 cell-specific suppressive activity (56, 57). First, T-bet mediates Treg migration: T-bet-deficient Tregs failed to upregulate CXCR3, which impaired their recruitment to sites of Th1 responses that were rich in its ligands CXCL9 and CXCL10 (36, 57). Second, T-bet selectively influences cytokine production: selective ablation of T-bet in Tregs suppressed IFN-γ production, but unrestrainedly produced Th2 and Th17 cell cytokines (56, 58). Of note, in certain condition, T-bet-deficient Tregs are equally suppressive as wide-type Tregs (59, 60), even have greater suppressive capacities (61). That is because T-bet^+^ Tregs were described to only specifically inhibit T-bet^+^ Teffs (58).

These characteristics of T-bet in Tregs are partly consistent with findings in the context of T1D. For example, the polymorphism of T-bet has been implicated as a risk gene in human T1D (62); in animals, T-bet was contributed to the control of diabetes in NOD mice; when lacking endogenous Tregs, those T-bet deficiency mice showed rapid progression of diabetes at early ages (63). These results suggested T-bet, combined with Tregs, was crucial for disease development in the context of T1D. Furthermore, T-bet-deficient Tregs displayed instability in the islet, failing to suppress CD4^+^ T cell and their infiltration in islet and PLNs (64).

However, T-bet failed to induce IFN-γ production in pancreatic CXCR3^+^ Tregs (21), which was coincident with T-bet function in other diseases (36). In addition, loss of T-bet by Tregs did not seem to affect Treg proliferation and survival in terms of overall numbers in pancreas, which considered to be compensating for impaired CXCR3-mediated migration into the islets (21). These results were consistent with the fact that T-bet^+^ Tregs differentiated from T-bet^−^ precursors rather than arising from an expansion of the steady-state T-bet^+^ Treg population (58). Thus, T-bet regulated CXCR3 expression in pancreatic-Tregs, which potentially contributed to disease progression in the context of T1D.

### Other Signal Pathways Mediated Tregs Expression and Function in Pancreatic Tissues and PLNs

Other signal pathways, such as IFN-γ/IFN-γR, TLR4/MD2, and IL-35, can also influence the expression and function of Tregs residing in pancreas or PLNs. Pancreatic-resident ICOS^+^ Tregs produced higher levels of IFN-γR and exhibited STAT1 phosphorylation upon stimulation, which suggested that ICOS^+^ Tregs were sensitive to IFN-γR signaling pathway (35). Consequently, IFN-γ selectively upregulated CXCR3 in ICOS^+^ Tregs *in vitro* and *in vivo* (35). It is speculated that IFN-γ produced by intra-islet Teffs might significantly upregulate CXCR3 in ICOS^+^ Tregs.

Another positive regulator of Tregs is IL-35, which is thought to maintain the phenotype of PLN Tregs (65), and ectopic expression of which reduced islet Foxp3^+^ Treg numbers and proliferation (66). In addition, pancreatic-Tregs could be induced using agonistic TLR4/MD-2 monoclonal antibody (Ab) (67). TLR4 signals have shown inconsistent effect (increased, decreased, and no effects) on T1D incidence (68–70), but one study has reported that agonistic TLR4/MD-2 Ab could substantially increase the number of Foxp3^+^Helios^+^Nrp-1^+^ Treg subset in pancreatic islet (67). Expansion of intra-islet Tregs, however, was not mediated by a direct effect of TLR-Ab on Tregs but was accompanied with resultant induction of tolerance of antigen-presenting cells (67), which supported the viewpoint of how TLR signals increased Tregs numbers (71). It should be noted that it is still unknown whether these above described signals have an exclusive capacity to modulate pancreatic-resident Tregs in the context of T1D.

Finally, pancreatic Tregs depend on TCR signals. TCR bound to peptide–MHC complexes, initiating signaling cascades that determined the stability and function of Tregs (72, 73), the process of which was also partly dependent on T cell-derived TGF-β and IL-2 (74). Indeed, lentivirus-mediated TCR gene transfer into polyclonal Treg could produce islet antigen-specific Treg populations, in which activation marker (CD69, CD137, and GARP) were upregulated (75). However, much effort is still needed to identify TCR diversity that contributes to the specific function of pancreatic Tregs in T1D.

To summarize, these factors might be used to target pancreatic-resident Tregs to expand Treg numbers or enhance their function or promote their migration into pancreas and PLN, thereby suppressing immune responses to prevent β cell destruction. It should be noted that whether there are distinct signal pathways that affect different Tregs subsets in pancreatic local settings is currently unknown, although recent studies indicate specific requirements do exist. Furthermore, identifying new specialized regulators might be particularly instructive for manipulating pancreatic-resident Tregs. Future studies to define how pancreatic Treg differentiation, homeostasis and function in the local settings will pave the way to manipulate tissue-resident Tregs for the treatment of T1D.

## Accumulation of Tregs in Pancreatic Tissues and PLNs

Regulatory T cells can generate in the thymus (termed thymic Treg, tTreg) and induce in the periphery by conversion of CD4^+^ Foxp3^−^ T cells into CD4^+^Foxp3^+^ Tregs (termed peripheral Treg, pTreg). Where and how T cells are developed into pancreatic-resident Tregs is currently unknown, although recent studies indicate some specific factors exist for their maintenance in pancreas. For example, CXCR3^+^ Tregs in pancreas are crucially dependent on transcription factor T-bet (21), possible positive feedback loop of IL-2–ICOS is important for pancreatic ICOS^+^ Treg homeostasis (36). Furthermore, the possibility remains that tTregs constitute a substantial proportion of pancreatic-resident Tregs. This hypothesis is supported by the fact that pancreatic CXCR3^+^ Tregs were regulated by T-bet (21), but this regulation was only observed in pancreatic natural Tregs (64). However, further investigation is required to comprehensively define the origin of pancreatic-resident Tregs that coordinate protective effects in T1D.

**Figure 1:**
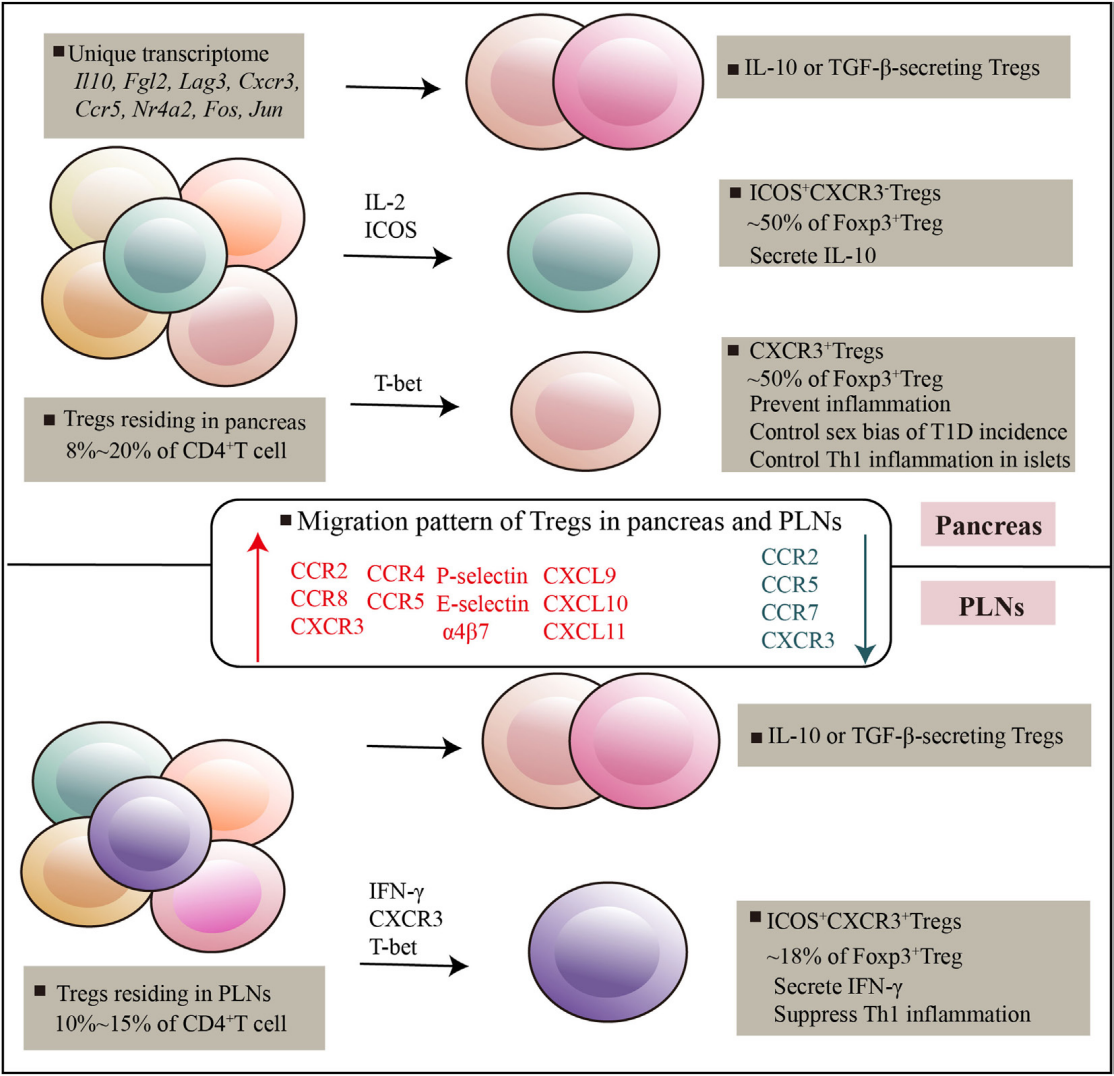
Regulatory T cells (Tregs) residing in pancreatic tissues and pancreatic lymph nodes. Pancreatic-resident Tregs had a transcriptome distinct from that of splenic counterparts, genes encoding cytokines and chemokines, and their receptors, and transcripton factors are highly expressed. There are at least three subsets can be distinguished on the basis of ICOS and CXCR3, which exert potent and distinguishable migratory and suppressive activity. Various immune mediators such as T-bet, IL-2, ICOS signals mediated Treg development and suppressive function in tissues. Specific receptors expressed by Tregs and specific chemotactic molecules produced by local environment promote Tregs recruitment and adaptation in local tissues.

The pattern of Treg accumulation in pancreas is governed by adhesion molecules, chemotactic molecules and chemoattractant receptors that expressed or produced by Tregs and pancreatic environmental cues (Figure 1). In the context of T1D, CXCR3^−^ Tregs show impaired accumulation in pancreatic islets. This effect has functional consequences, as mice defective in CXCR3 could not effectively suppress Th1 activity and developed earlier onset of diabetes (76). Besides CXCR3, pancreatic Tregs express several other chemokine receptors such as CCR2, CCR8, and CXCR6, which were thought to compensate for the absence of CXCR3 in promoting Tregs localization to the islets. For example, early studies described the migration pattern of Tregs in transplanted pancreatic islets and showed that Tregs needed to be educated first in the inflamed islets before entering the draining lymph node (77). Tregs migrated from blood to the inflamed islets depending on CCR2, CCR4, CCR5, and P- and E-selectin ligands, whereas Tregs migrated to draining lymph nodes in a CCR2, CCR5, and CCR7 fashion (77). Other molecules such as CCL12 and MAdCAM-1 expressed by islet, SDF and Integrin α4β7 expressed by Tregs were required for Treg localization in the pancreas (16, 78–80). Furthermore, pancreatic-resident antigen-presenting cells, specifically F4/80^+^ macrophages and CD11c^+^ DCs, were commonly expressed CXCL9, CXCL10, and CXCL11, which specifically induced CXCR3^+^ Tregs migration in islets (35). These data suggest that substantial migration is required for Treg suppressive function in local setting, this process involves in an array of molecules that act in a redundant fashion. No detailed insights exist as to which of these molecules are more important for Treg migration between pancreas and PLNs. It is also not clear whether these migration patterns performed by pancreatic Tregs are primarily in the context of T1D or any other inflammatory responses.

Transcriptional factors were also observed to affect Treg migration in T1D. T-bet^−/−^ natural Treg expressed more CCR4 and migrated better to CCL22, which contributed to T-bet^−/−^ Treg in the islet (64, 77). Also, T-bet affected expression of sphingosine-1-phosphate (S1P) receptor 1 (81), which bonded to S1P and promoted migration of Tregs from tissues into the blood (82). Thereby, T-bet deficiency resulted in a defective migration of Tregs across lymphatic endothelial cells and was accompanied by retaining T-bet^−/−^ Treg in pancreatic tissues (81). Similarly, CD103 (also known as αE integrin) expression can be influenced by T-bet (36, 64). CD103 formed a heterodimer with β7 integrin and contributed to specific retention of lymphocytes in tissues by binding to E-cadherin on epithelial cells (83, 84). CD103 has been used to identify tissue-resident lymphocytes (85); hence, CD103 upregulated by T-bet deficiency was functionally involved in retaining T-bet^−/−^ Tregs in islets (64). In addition, as described above, T-bet induced CXCR3 expression on Tregs, which was prone to residing in pancreas (76). Altogether, chemokine and chemokine receptors are regulated by T-bet in Tregs, and their function in terms of ligand interactions indicate that T-bet is involved in the retention of Tregs in pancreatic tissues.

It should be noted that mechanisms underlying Treg migration are shared with those of pathogenic effector cells. For example, α4β7 was highly expressed on islet-infiltrating lymphocytes in NOD mice, suggesting that the MAdCAM-1 in inflamed islets also drove the migration of pathogenic Teffs into islets (78, 86). It has been described that immunocytes moved into the pancreas in a dynamic manner during disease progression. Insulitic lesion was continuously infiltrated by a mixed influx of immunocytes: both naïve- and memory-phenotype lymphocytes could traffic to the insulitis, but Tregs had less active migration than their conventional CD4^+^ counterparts (87). Thus, there is a lot of interest to know when and how preferential recruitment of Tregs was happened in pancreas in the context of T1D. A more complete understanding of the detailed migration mechanism that is necessary for Treg accumulation in pancreas remains to be elucidated and will be important for expansion and development of therapeutic Tregs in response to the tissue-specific environment.

## Non-Immunological Functions of Tregs in Pancreatic Tissues and PLNs

Regulatory T cells potently suppress the activation and function of other immune cells through directly cell-to-cell contact or indirectly secreted factors. In addition to regulate immunological process, growing evidence showed that Tregs, particularly tissue-resident Tregs, also regulated non-immunological functions, which has been well studied in muscle and visceral adipose tissue. Thus, in the context of T1D, non-traditional roles should be considered and evaluated as an important facet of pancreatic-resident Tregs.

Although there are no data so far to directly show non-immunological role of pancreatic-resident Tregs, there is evidence suggesting a role for Treg-derived mediators in regulating β cells. First, ICOS^+^ Tregs expressed IFN-γR at higher levels and prevented IFN-γ production. Insulin^+^ β cells expressing IFN-γR could respond to IFN-γ, thereby, promoting apoptosis of islet cells. Thus, Tregs may have an indirect role to prevent the destruction of the islets (35, 88). Second, in PLNs CXCR4/SDF-1 axis was involved in retaining Tregs, these molecules also contributed to the regeneration of autologous β cells in the antea-diabetic model (16). Third, TGF-β has been viewed as a protective cytokine in β cell mass, and consequently, TGF-β signaling has an important role in pancreatic development and islet homeostasis (89–91). However, a recent study described that inhibition of TGF-β pathway promoted β cell replication, which suggests that Tregs-secreted TGF-β might have a detriment effect on maintenance and expansion of β cell mass (91). This study found that TGF-β signaling induced age-related accumulation of p16^INK4a^ that led to pancreatic β-cell proliferation decline; by contrast, treatment with TGF-β inhibitor exhibited a significant replication of β cells in both mouse and human (91). Although less well characterized, based on our current understanding, it is plausible that Tregs that are enriched in the pancreas may contribute to protecting β cells at least in part by non-immune-mediated ways.

However, these studies raise the question whether pancreatic-resident Tregs could secrete some factors that specifically target to β cell and promote their repair and regeneration. These tissue-specific factors will allow Tregs to reside in pancreatic tissues and act appropriate effector function in response to β cell destruction. However, given the distinct pancreatic-resident Treg subsets already found in T1D, it seems that their primary role is to prevent β cell destruction by locally restraining inflammatory responses of both innate and adaptive immune cells (49, 92–94).

## Perspective and Conclusion

The identification of pancreatic-resident Tregs and recognition of their diverse phenotype and potent immunosuppressive activity have provided new insights into how Tregs function in tissue-specific environment and why the results of manipulating Tregs for the treatment of T1D have been disappointing in some cases. Although present in pancreas in relatively low numbers, the selectively higher proportion of some unique subsets in pancreatic infiltration and PLNs seems to confer a remarkable ability to regulate physiological process during T1D. Thus, the ability of pancreatic-resident Tregs to hamper β cell destruction clearly emphasizes its use as an immunotherapeutic strategy in T1D. Tailoring proliferation and apoptosis of these Tregs in response to pancreatic-specific inflammatory milieu would promote immune balance, allowing for preservation of remaining β cells in T1D. Indeed, islet-specific IL-2 has proved beneficial in the treatment of NOD mice, and increased pancreatic-resident Treg numbers (55). However, as the study of pancreatic-resident Tregs is a new field, many questions regarding their properties remain unaddressed.

Given the diversity of tissue-resident Tregs already found, transcriptional factors that are specific to corresponding tissues are contributed to their non-immunological activity, for example amphiregulin in the muscle, PPAR-γ in the VAT. The importance of this property failed to describe in the pancreatic-resident Tregs. In contrast to pancreatic tissues, tissues that already found specific-Tregs are under constant threat of invasion and prone to damage. It might be that islet surrounding microenvironment did not support the specific functions of Tregs, as individual tissues have unique challenges and immune response. It is also difficult to rule out the possibility that such novel Tregs residing in pancreas are unrecognized. It is reminiscent of the finding that BLIMP-1- and IRF4-dependent Treg signatures were biased in pancreatic Tregs compared with spleen Tregs, but their function has not been assessed (21). These two transcription factors BLIMP-1 and IRF4 have been reported for differentiation and function of effector Tregs in tissue homeostasis (95). Thus, future studies must identify the precise regulators of Tregs that mediates functional difference in the pancreatic microenvironment, knowing this will probably reflect pathophysiological complexity and therapeutic targets of T1D.

Despite evidence has revealed an important role of pancreatic-resident Tregs in T1D mice, very few current studies have performed detailed investigations of these cells in patients with T1D. Human CD4^+^ and CD8^+^ T cells in islets lack expression of CD25 in their healthy state (96), and only ~5% Tregs can be found in PLNs of T1D (24). In addition, limited knowledge is available with respect to immunosuppressive effector of pancreatic-Tregs, although functional defects in Tregs have been partly characterized by analysis of the Treg-specific demethylated region within the Foxp3 locus in PLN of T1D patients (9). These data raise the question whether Tregs recruit in pancreatic tissues only in physiological state in human. And the hypotheses that have aimed to ascribe human T1D pathophysiology to the defect of pancreatic-Tregs are confounded by our limited understanding of Treg population within the islet. Thus, a more complete understanding of features of human pancreatic-Tregs would be important for evaluating current efforts to target human Tregs with intra-islet in the context of T1D.

Finally, the question remains how important of pancreatic Tregs is for T1D development. The immune systems have evolved so that different cell subsets with specialized function collaborate to influence diabetes progression; therefore, it is important to consider dominant populations in this process. So far, the ability of pancreatic-Tregs to delay the onset of diabetes has been seen as a sign of superior function, but this phenotype could also represent systematic Tregs. In addition, it remains unclear how the pancreatic-Tregs modify immune responses by cooperating with immune cells of both the innate and adaptive immune systems. Increased in-depth understanding of these issues discussed above is need for the better development of therapeutic interventions in T1D.

## Author Contributions

JL, CZ, XZ, and LL participated in compilation and editing of the manuscript. JL, CZ, LL, XZ, WX, and CZ participated in literature and data collection. All authors read and approved the final version.

## Conflict of Interest Statement

The authors declare that the research was conducted in the absence of any commercial or financial relationships that could be construed as a potential conflict of interest.

## References

[1] Lehuen A Diana J Zaccone P Cooke A. Immune cell crosstalk in type 1 diabetes. Nat Rev Immunol (2010) 10(7):501–13. 10.1038/nri2787 20577267

[2] Fraker C Bayer AL The expanding role of natural killer cells in type 1 diabetes and immunotherapy. Curr Diab Rep (2016) 16(11):109 10.1007/s11892-016-0806-7 27664042

[3] Morel PA. Dendritic cell subsets in type 1 diabetes: friend or foe? Front Immunol (2013) 4:415. 10.3389/fimmu.2013.00415 24367363 PMC3853773

[4] Lu L Barbi J Pan F. The regulation of immune tolerance by FOXP3. Nat Rev Immunol (2017). 10.1038/nri.2017.75 28757603 PMC5793224

[5] Salomon B Lenschow DJ Rhee L Ashourian N Singh B Sharpe A B7/CD28 costimulation is essential for the homeostasis of the CD4+CD25+ immunoregulatory T cells that control autoimmune diabetes. Immunity (2000) 12(4):431–40. 10.1016/S1074-7613(00)80195-8 10795741

[6] Rosenzwajg M Churlaud G Mallone R Six A Derian N Chaara W Low-dose interleukin-2 fosters a dose-dependent regulatory T cell tuned milieu in T1D patients. J Autoimmun (2015) 58:48–58. 10.1016/j.jaut.2015.01.001 25634360 PMC8153751

[7] Marek-Trzonkowska N Mysliwiec M Dobyszuk A Grabowska M Techmanska I Juscinska J Administration of CD4+CD25highCD127- regulatory T cells preserves beta-cell function in type 1 diabetes in children. Diabetes Care (2012) 35(9):1817–20. 10.2337/dc12-0038 22723342 PMC3425004

[8] Marek-Trzonkowska N Mysliwiec M Dobyszuk A Grabowska M Derkowska I Juscinska J Therapy of type 1 diabetes with CD4(+)CD25(high)CD127-regulatory T cells prolongs survival of pancreatic islets – results of one year follow-up. Clin Immunol (2014) 153(1):23–30. 10.1016/j.clim.2014.03.016 24704576

[9] Ferraro A Socci C Stabilini A Valle A Monti P Piemonti L Expansion of Th17 cells and functional defects in T regulatory cells are key features of the pancreatic lymph nodes in patients with type 1 diabetes. Diabetes (2011) 60(11):2903–13. 10.2337/db11-0090 21896932 PMC3198077

[10] Pesenacker AM Wang AY Singh A Gillies J Kim Y Piccirillo CA A regulatory T-cell gene signature is a specific and sensitive biomarker to identify children with new-onset type 1 diabetes. Diabetes (2016) 65(4):1031–9. 10.2337/db15-0572 26786322

[11] Zoka A Barna G Somogyi A Muzes G Olah A Al-Aissa Z Extension of the CD4(+)Foxp3(+)CD25(-/low) regulatory T-cell subpopulation in type 1 diabetes mellitus. Autoimmunity (2015) 48(5):289–97. 10.3109/08916934.2014.992518 25523632

[12] Lindley S Dayan CM Bishop A Roep BO Peakman M Tree TI. Defective suppressor function in CD4(+)CD25(+) T-cells from patients with type 1 diabetes. Diabetes (2005) 54(1):92–9. 10.2337/diabetes.54.1.92 15616015

[13] Brusko T Wasserfall C McGrail K Schatz R Viener HL Schatz D No alterations in the frequency of FOXP3+ regulatory T-cells in type 1 diabetes. Diabetes (2007) 56(3):604–12. 10.2337/db06-1248 17327427

[14] Burzyn D Benoist C Mathis D. Regulatory T cells in nonlymphoid tissues. Nat Immunol (2013) 14(10):1007–13. 10.1038/ni.2683 24048122 PMC4708287

[15] Panduro M Benoist C Mathis D Tissue Tregs. Annu Rev Immunol (2016) 34:609–33. 10.1146/annurev-immunol-032712-095948 27168246 PMC4942112

[16] Nti BK Markman JL Bertera S Styche AJ Lakomy RJ Subbotin VM Treg cells in pancreatic lymph nodes: the possible role in diabetogenesis and beta cell regeneration in a T1D model. Cell Mol Immunol (2012) 9(6):455–63. 10.1038/cmi.2012.36 23042535 PMC4002217

[17] Sebastiani G Ventriglia G Stabilini A Socci C Morsiani C Laurenzi A Regulatory T-cells from pancreatic lymphnodes of patients with type-1 diabetes express increased levels of microRNA miR-125a-5p that limits CCR2 expression. Sci Rep (2017) 7(1):6897. 10.1038/s41598-017-07172-1 28761107 PMC5537269

[18] Tanoue T Atarashi K Honda K. Development and maintenance of intestinal regulatory T cells. Nat Rev Immunol (2016) 16(5):295–309. 10.1038/nri.2016.36 27087661

[19] Cipolletta D Feuerer M Li A Kamei N Lee J Shoelson SE PPAR-γ is a major driver of the accumulation and phenotype of adipose tissue T_reg_ cells. Nature (2012) 486(7404):549–53. 10.1038/nature11132 22722857 PMC3387339

[20] Grinberg-Bleyer Y Baeyens A You S Elhage R Fourcade G Gregoire S IL-2 reverses established type 1 diabetes in NOD mice by a local effect on pancreatic regulatory T cells. J Exp Med (2010) 207(9):1871–8. 10.1084/jem.20100209 20679400 PMC2931175

[21] Tan TG Mathis D Benoist C. Singular role for T-BET+CXCR3+ regulatory T cells in protection from autoimmune diabetes. Proc Natl Acad Sci U S A (2016) 113(49):14103–8. 10.1073/pnas.1616710113 27872297 PMC5150376

[22] Mellanby RJ Thomas D Phillips JM Cooke A Diabetes in non-obese diabetic mice is not associated with quantitative changes in CD4+ CD25+ Foxp3+ regulatory T cells. Immunology (2007) 121(1):15–28. 10.1111/j.1365-2567.2007.02546.x 17428252 PMC2265922

[23] Herman AE Freeman GJ Mathis D Benoist C. CD4+CD25+ T regulatory cells dependent on ICOS promote regulation of effector cells in the prediabetic lesion. J Exp Med (2004) 199(11):1479–89. 10.1084/jem.20040179 15184501 PMC2211778

[24] Seay HR Yusko E Rothweiler SJ Zhang L Posgai AL Campbell-Thompson M Tissue distribution and clonal diversity of the T and B cell repertoire in type 1 diabetes. JCI insight (2016) 1(20):e88242. 10.1172/jci.insight.88242 27942583 PMC5135280

[25] Dong C Nurieva RI Prasad DV. Immune regulation by novel costimulatory molecules. Immunol Res (2003) 28(1):39–48. 10.1385/IR:28:1:39 12947223

[26] Nurieva RI Duong J Kishikawa H Dianzani U Rojo JM Ho I Transcriptional regulation of th2 differentiation by inducible costimulator. Immunity (2003) 18(6):801–11. 10.1016/S1074-7613(03)00144-4 12818161

[27] Kornete M Sgouroudis E Piccirillo CA. ICOS-dependent homeostasis and function of Foxp3+ regulatory T cells in islets of nonobese diabetic mice. J Immunol (2012) 188(3):1064–74. 10.4049/jimmunol.1101303 22227569

[28] Ito T Hanabuchi S Wang YH Park WR Arima K Bover L Two functional subsets of FOXP3+ regulatory T cells in human thymus and periphery. Immunity (2008) 28(6):870–80. 10.1016/j.immuni.2008.03.018 18513999 PMC2709453

[29] Kohyama M Sugahara D Sugiyama S Yagita H Okumura K Hozumi N. Inducible costimulator-dependent IL-10 production by regulatory T cells specific for self-antigen. Proc Natl Acad Sci U S A (2004) 101(12):4192–7. 10.1073/pnas.0400214101 15014176 PMC384717

[30] McAdam AJ Chang TT Lumelsky AE Greenfield EA Boussiotis VA Duke-Cohan JS Mouse inducible costimulatory molecule (ICOS) expression is enhanced by CD28 costimulation and regulates differentiation of CD4+ T cells. J Immunol (2000) 165(9):5035–40. 10.4049/jimmunol.165.9.5035 11046032

[31] Lohning M Hutloff A Kallinich T Mages HW Bonhagen K Radbruch A Expression of ICOS in vivo defines CD4+ effector T cells with high inflammatory potential and a strong bias for secretion of interleukin 10. J Exp Med (2003) 197(2):181–93. 10.1084/jem.20020632 12538658 PMC2193816

[32] Warnatz K Bossaller L Salzer U Skrabl-Baumgartner A Schwinger W van der Burg M Human ICOS deficiency abrogates the germinal center reaction and provides a monogenic model for common variable immunodeficiency. Blood (2006) 107(8):3045–52. 10.1182/blood-2005-07-2955 16384931

[33] Vocanson M Rozieres A Hennino A Poyet G Gaillard V Renaudineau S Inducible costimulator (ICOS) is a marker for highly suppressive antigen-specific T cells sharing features of TH17/TH1 and regulatory T cells. J Allergy Clin Immunol (2010) 126(2):280–9. 9 e1-7, 10.1016/j.jaci.2010.05.022 20624644

[34] Yagi J Arimura Y Dianzani U Uede T Okamoto T Uchiyama T. Regulatory roles of IL-2 and IL-4 in H4/inducible costimulator expression on activated CD4+ T cells during Th cell development. J Immunol (2003) 171(2):783–94. 10.4049/jimmunol.171.2.783 12847246

[35] Kornete M Mason ES Girouard J Lafferty EI Qureshi S Piccirillo CA Th1-like ICOS+ Foxp3+ Treg cells preferentially express CXCR3 and home to beta-islets during pre-diabetes in BDC2.5 NOD mice. PLoS One (2015) 10(5):e0126311 10.1371/journal.pone.0126311 25946021 PMC4422433

[36] Koch MA Tucker-Heard G Perdue NR Killebrew JR Urdahl KB Campbell DJ. The transcription factor T-bet controls regulatory T cell homeostasis and function during type 1 inflammation. Nat Immunol (2009) 10(6):595–602. 10.1038/ni.1731 19412181 PMC2712126

[37] Brodin P Davis MM. Human immune system variation. Nat Rev Immunol (2017) 17(1):21–9. 10.1038/nri.2016.125 27916977 PMC5328245

[38] Green EA Gorelik L McGregor CM Tran EH Flavell RA. CD4+CD25+ T regulatory cells control anti-islet CD8+ T cells through TGF-beta-TGF-beta receptor interactions in type 1 diabetes. Proc Natl Acad Sci U S A (2003) 100(19):10878–83. 10.1073/pnas.1834400100 12949259 PMC196896

[39] Gutcher I Donkor MK Ma Q Rudensky AY Flavell RA Li MO Autocrine transforming growth factor-beta1 promotes in vivo Th17 cell differentiation. Immunity (2011) 34(3):396–408. 10.1016/j.immuni.2011.03.005 21435587 PMC3690311

[40] Li MO Wan YY Flavell RA T cell-produced transforming growth factor-beta1 controls T cell tolerance and regulates Th1- and Th17-cell differentiation. Immunity (2007) 26(5):579–91. 10.1016/j.immuni.2007.03.014 17481928

[41] Ishigame H Zenewicz LA Sanjabi S Licona-Limon P Nakayama M Leonard WJ Excessive Th1 responses due to the absence of TGF-beta signaling cause autoimmune diabetes and dysregulated Treg cell homeostasis. Proc Natl Acad Sci U S A (2013) 110(17):6961–6. 10.1073/pnas.1304498110 23569233 PMC3637710

[42] Li C Zhang L Chen Y Lin X Li T Protective role of adenovirus vector-mediated interleukin-10 gene therapy on endogenous islet beta-cells in recent-onset type 1 diabetes in NOD mice. Exp Ther Med (2016) 11(5):1625–32. 10.3892/etm.2016.3169 27168782 PMC4840566

[43] Saraiva M O’Garra A. The regulation of IL-10 production by immune cells. Nat Rev Immunol (2010) 10(3):170–81. 10.1038/nri2711 20154735

[44] Thorsen SU Pipper CB Eising S Skogstrand K Hougaard DM Svensson J Neonatal levels of adiponectin, interleukin-10 and interleukin-12 are associated with the risk of developing type 1 diabetes in childhood and adolescence: a nationwide Danish case-control study. Clin Immunol (2017) 174:18–23. 10.1016/j.clim.2016.11.007 27871914

[45] Cheng G Yu A Dee MJ Malek TR. IL-2R signaling is essential for functional maturation of regulatory T cells during thymic development. J Immunol (2013) 190(4):1567–75. 10.4049/jimmunol.1201218 23315074 PMC3563871

[46] Boyman O Sprent J. The role of interleukin-2 during homeostasis and activation of the immune system. Nat Rev Immunol (2012) 12(3):180–90. 10.1038/nri3156 22343569

[47] Yamanouchi J Rainbow D Serra P Howlett S Hunter K Garner VE Interleukin-2 gene variation impairs regulatory T cell function and causes autoimmunity. Nat Genet (2007) 39(3):329–37. 10.1038/ng1958 17277778 PMC2886969

[48] Ghosh S Palmer SM Rodrigues NR Cordell HJ Hearne CM Cornall RJ Polygenic control of autoimmune diabetes in nonobese diabetic mice. Nat Genet (1993) 4(4):404–9. 10.1038/ng0893-404 8401590

[49] Tang Q Adams JY Penaranda C Melli K Piaggio E Sgouroudis E Central role of defective interleukin-2 production in the triggering of islet autoimmune destruction. Immunity (2008) 28(5):687–97. 10.1016/j.immuni.2008.03.016 18468463 PMC2394854

[50] Manirarora JN Wei CH. Combination therapy using IL-2/IL-2 monoclonal antibody complexes, rapamycin, and islet autoantigen peptides increases regulatory T cell frequency and protects against spontaneous and induced type 1 diabetes in nonobese diabetic mice. J Immunol (2015) 195(11):5203–14. 10.4049/jimmunol.1402540 26482409

[51] Hartemann A Bensimon G Payan CA Jacqueminet S Bourron O Nicolas N Low-dose interleukin 2 in patients with type 1 diabetes: a phase 1/2 randomised, double-blind, placebo-controlled trial. Lancet Diabetes Endocrinol (2013) 1(4):295–305. 10.1016/S2213-8587(13)70113-X 24622415

[52] Todd JA Evangelou M Cutler AJ Pekalski ML Walker NM Stevens HE Regulatory T cell responses in participants with type 1 diabetes after a single dose of interleukin-2: a non-randomised, open label, adaptive dose-finding trial. PLoS Med (2016) 13(10):e1002139. 10.1371/journal.pmed.1002139 27727279 PMC5058548

[53] Zheng SG Wang J Wang P Gray JD Horwitz DA. IL-2 is essential for TGF-beta to convert naive CD4+CD25- cells to CD25+Foxp3+ regulatory T cells and for expansion of these cells. J Immunol (2007) 178(4):2018–27. 10.4049/jimmunol.178.4.2018 17277105

[54] Tang Q Therapeutic window of interleukin-2 for autoimmune diseases. Diabetes (2015) 64(6):1912–3. 10.2337/db15-0188 25999537 PMC4439560

[55] Johnson MC Garland AL Nicolson SC Li C Samulski RJ Wang B beta-cell-specific IL-2 therapy increases islet Foxp3+Treg and suppresses type 1 diabetes in NOD mice. Diabetes (2013) 62(11):3775–84. 10.2337/db13-0669 23884888 PMC3806588

[56] Nosko A Kluger MA Diefenhardt P Melderis S Wegscheid C Tiegs G T-bet enhances regulatory T cell fitness and directs control of th1 responses in crescentic GN. J Am Soc Nephrol (2017) 28(1):185–96. 10.1681/ASN.2015070820 27297951 PMC5198267

[57] McPherson RC Turner DG Mair I O’Connor RA Anderton SM. T-bet expression by Foxp3(+) T regulatory cells is not essential for their suppressive function in CNS autoimmune disease or colitis. Front Immunol (2015) 6:69. 10.3389/fimmu.2015.00069 25741342 PMC4332357

[58] Levine AG Medoza A Hemmers S Moltedo B Niec RE Schizas M Stability and function of regulatory T cells expressing the transcription factor T-bet. Nature (2017) 546(7658):421–5. 10.1038/nature22360 28607488 PMC5482236

[59] Bettelli E Sullivan B Szabo SJ Sobel RA Glimcher LH Kuchroo VK. Loss of T-bet, but not STAT1, prevents the development of experimental autoimmune encephalomyelitis. J Exp Med (2004) 200(1):79–87. 10.1084/jem.20031819 15238607 PMC2213316

[60] Finotto S Hausding M Doganci A Maxeiner JH Lehr HA Luft C Asthmatic changes in mice lacking T-bet are mediated by IL-13. Int Immunol (2005) 17(8):993–1007. 10.1093/intimm/dxh281 16000330

[61] Neurath MF Weigmann B Finotto S Glickman J Nieuwenhuis E Iijima H The transcription factor T-bet regulates mucosal T cell activation in experimental colitis and Crohn’s disease. J Exp Med (2002) 195(9):1129–43. 10.1084/jem.20011956 11994418 PMC2193714

[62] Sasaki Y Ihara K Matsuura N Kohno H Nagafuchi S Kuromaru R Identification of a novel type 1 diabetes susceptibility gene, T-bet. Human Genet (2004) 115(3):177–84. 10.1007/s00439-004-1146-2 15241679

[63] Esensten JH Lee MR Glimcher LH Bluestone JA. T-bet-deficient NOD mice are protected from diabetes due to defects in both T cell and innate immune system function. J Immunol (2009) 183(1):75–82. 10.4049/jimmunol.0804154 19535634 PMC2732575

[64] Xiong Y Ahmad S Iwami D Brinkman CC Bromberg JS. T-bet regulates natural regulatory T cell afferent lymphatic migration and suppressive function. J Immunol (2016) 196(6):2526–40. 10.4049/jimmunol.1502537 26880765 PMC4779695

[65] Singh K Kadesjo E Lindroos J Hjort M Lundberg M Espes D Interleukin-35 administration counteracts established murine type 1 diabetes – possible involvement of regulatory T cells. Sci Rep (2015) 5:12633 10.1038/srep12633 26224624 PMC4519737

[66] Manzoor F Johnson MC Li C Samulski RJ Wang B Tisch R beta-cell-specific IL-35 therapy suppresses ongoing autoimmune diabetes in NOD mice. Eur J Immunol (2017) 47(1):144–54. 10.1002/eji.201646493 27859048 PMC5233468

[67] Bednar KJ Tsukamoto H Kachapati K Ohta S Wu Y Katz JD Reversal of new-onset type 1 diabetes with an agonistic TLR4/MD-2 monoclonal antibody. Diabetes (2015) 64(10):3614–26. 10.2337/db14-1868 26130764 PMC9162148

[68] Gulden E Ihira M Ohashi A Reinbeck AL Freudenberg MA Kolb H Toll-like receptor 4 deficiency accelerates the development of insulin-deficient diabetes in non-obese diabetic mice. PLoS One (2013) 8(9):e75385. 10.1371/journal.pone.0075385 24086519 PMC3781027

[69] Wen L Ley RE Volchkov PY Stranges PB Avanesyan L Stonebraker AC Innate immunity and intestinal microbiota in the development of Type 1 diabetes. Nature (2008) 455(7216):1109–13. 10.1038/nature07336 18806780 PMC2574766

[70] Devaraj S Tobias P Jialal I. Knockout of toll-like receptor-4 attenuates the pro-inflammatory state of diabetes. Cytokine (2011) 55(3):441–5. 10.1016/j.cyto.2011.03.023 21498084

[71] Sutmuller RP Morgan ME Netea MG Grauer O Adema GJ. Toll-like receptors on regulatory T cells: expanding immune regulation. Trends Immunol (2006) 27(8):387–93. 10.1016/j.it.2006.06.005 16814607

[72] Hawse WF Boggess WC Morel PA TCR signal strength regulates Akt substrate specificity to induce alternate murine Th and T regulatory cell differentiation programs. J Immunol (2017) 199(2):589–97. 10.4049/jimmunol.1700369 28600288 PMC5575766

[73] Huynh A DuPage M Priyadharshini B Sage PT Quiros J Borges CM Control of PI(3) kinase in Treg cells maintains homeostasis and lineage stability. Nat Immunol (2015) 16(2):188–96. 10.1038/ni.3077 25559257 PMC4297515

[74] Turner MS Isse K Fischer DK Turnquist HR Morel PA. Low TCR signal strength induces combined expansion of Th2 and regulatory T cell populations that protect mice from the development of type 1 diabetes. Diabetologia (2014) 57(7):1428–36. 10.1007/s00125-014-3233-9 24737163

[75] Hull CM Nickolay LE Estorninho M Richardson MW Riley JL Peakman M Generation of human islet-specific regulatory T cells by TCR gene transfer. J Autoimmun (2017) 79:63–73. 10.1016/j.jaut.2017.01.001 28117148

[76] Yamada Y Okubo Y Shimada A Oikawa Y Yamada S Narumi S Acceleration of diabetes development in CXC chemokine receptor 3 (CXCR3)-deficient NOD mice. Diabetologia (2012) 55(8):2238–45. 10.1007/s00125-012-2547-8 22487925

[77] Zhang N Schroppel B Lal G Jakubzick C Mao X Chen D Regulatory T cells sequentially migrate from inflamed tissues to draining lymph nodes to suppress the alloimmune response. Immunity (2009) 30(3):458–69. 10.1016/j.immuni.2008.12.022 19303390 PMC2737741

[78] Yang XD Sytwu HK McDevitt HO Michie SA. Involvement of beta 7 integrin and mucosal addressin cell adhesion molecule-1 (MAdCAM-1) in the development of diabetes in obese diabetic mice. Diabetes (1997) 46(10):1542–7. 10.2337/diabetes.46.10.1542 9313747

[79] Weber SE Harbertson J Godebu E Mros GA Padrick RC Carson BD Adaptive islet-specific regulatory CD4 T cells control autoimmune diabetes and mediate the disappearance of pathogenic Th1 cells in vivo. J Immunol (2006) 176(8):4730–9. 10.4049/jimmunol.176.8.4730 16585566

[80] Montane J Bischoff L Soukhatcheva G Dai DL Hardenberg G Levings MK Prevention of murine autoimmune diabetes by CCL22-mediated Treg recruitment to the pancreatic islets. J Clin Invest (2011) 121(8):3024–8. 10.1172/JCI43048 21737880 PMC3148722

[81] Ledgerwood LG Lal G Zhang N Garin A Esses SJ Ginhoux F The sphingosine 1-phosphate receptor 1 causes tissue retention by inhibiting the entry of peripheral tissue T lymphocytes into afferent lymphatics. Nat Immunol (2008) 9(1):42–53. 10.1038/ni1534 18037890

[82] Shiow LR Rosen DB Brdickova N Xu Y An J Lanier LL CD69 acts downstream of interferon-alpha/beta to inhibit S1P1 and lymphocyte egress from lymphoid organs. Nature (2006) 440(7083):540–4. 10.1038/nature04606 16525420

[83] Hadley GA Higgins JM Integrin alphaEbeta7: molecular features and functional significance in the immune system. Adv Exp Med Biol (2014) 819:97–110. 10.1007/978-94-017-9153-3_7 25023170

[84] Cepek KL Shaw SK Parker CM Russell GJ Morrow JS Rimm DL Adhesion between epithelial cells and T lymphocytes mediated by E-cadherin and the alpha E beta 7 integrin. Nature (1994) 372(6502):190–3. 10.1038/372190a0 7969453

[85] Bjorkstrom NK Ljunggren HG Michaelsson J. Emerging insights into natural killer cells in human peripheral tissues. Nat Rev Immunol (2016) 16(5):310–20. 10.1038/nri.2016.34 27121652

[86] Hanninen A Taylor C Streeter PR Stark LS Sarte JM Shizuru JA Vascular addressins are induced on islet vessels during insulitis in nonobese diabetic mice and are involved in lymphoid cell binding to islet endothelium. J Clin Invest (1993) 92(5):2509–15. 10.1172/JCI116859 7693764 PMC288436

[87] Magnuson AM Thurber GM Kohler RH Weissleder R Mathis D Benoist C. Population dynamics of islet-infiltrating cells in autoimmune diabetes. Proc Natl Acad Sci U S A (2015) 112(5):1511–6. 10.1073/pnas.1423769112 25605891 PMC4321317

[88] Kim S Kim HS Chung KW Oh SH Yun JW Im SH Essential role for signal transducer and activator of transcription-1 in pancreatic beta-cell death and autoimmune type 1 diabetes of nonobese diabetic mice. Diabetes (2007) 56(10):2561–8. 10.2337/db06-1372 17620422

[89] Lin HM Lee JH Yadav H Kamaraju AK Liu E Zhigang D Transforming growth factor-beta/Smad3 signaling regulates insulin gene transcription and pancreatic islet beta-cell function. J Biol Chem (2009) 284(18):12246–57. 10.1074/jbc.M805379200 19265200 PMC2673293

[90] Xiao X Wiersch J El-Gohary Y Guo P Prasadan K Paredes J TGFbeta receptor signaling is essential for inflammation-induced but not beta-cell workload-induced beta-cell proliferation. Diabetes (2013) 62(4):1217–26. 10.2337/db12-1428 23248173 PMC3609557

[91] Dhawan S Dirice E Kulkarni RN Bhushan A Inhibition of TGF-beta signaling promotes human pancreatic beta-cell replication. Diabetes (2016) 65(5):1208–18. 10.2337/db15-1331 26936960 PMC4839200

[92] Tritt M Sgouroudis E d’Hennezel E Albanese A Piccirillo CA. Functional waning of naturally occurring CD4+ regulatory T-cells contributes to the onset of autoimmune diabetes. Diabetes (2008) 57(1):113–23. 10.2337/db06-1700 17928397

[93] Chen Z Herman AE Matos M Mathis D Benoist C. Where CD4+CD25+ T reg cells impinge on autoimmune diabetes. J Exp Med (2005) 202(10):1387–97. 10.1084/jem.20051409 16301745 PMC2212985

[94] Sitrin J Ring A Garcia KC Benoist C Mathis D. Regulatory T cells control NK cells in an insulitic lesion by depriving them of IL-2. J Exp Med (2013) 210(6):1153–65. 10.1084/jem.20122248 23650440 PMC3674700

[95] Cretney E Xin A Shi W Minnich M Masson F Miasari M The transcription factors Blimp-1 and IRF4 jointly control the differentiation and function of effector regulatory T cells. Nat Immunol (2011) 12(4):304–11. 10.1038/ni.2006 21378976

[96] Radenkovic M Uvebrant K Skog O Sarmiento L Avartsson J Storm P Characterization of resident lymphocytes in human pancreatic islets. Clin Exp Immunol (2017) 187(3):418–27. 10.1111/cei.12892 27783386 PMC5290249

